# Peanut allergy burden survey: Factors associated with health‐related quality of life in adolescents

**DOI:** 10.1002/clt2.12234

**Published:** 2023-03-26

**Authors:** William A. McCann, Anna Nowak‐Wegrzyn, Steven L. Hass, Danmeng Huang, Sarah M. Donelson

**Affiliations:** ^1^ Allergy Partners P.A. Asheville North Carolina USA; ^2^ NYU Grossman School of Medicine Hassenfeld Children's Hospital New York New York USA; ^3^ Department of Pediatrics, Gastroenterology and Nutrition Collegium Medicum University of Warmia and Mazury Olsztyn Poland; ^4^ H. E. Outcomes LLC Simi Valley Los Angeles California USA; ^5^ Xcenda LLC Carrolton Texas USA; ^6^ Aimmune Therapeutics A Nestlé Health Science Company Brisbane California USA

**Keywords:** adolescents, burden, food allergy, health‐related quality of life, peanut allergy

## Abstract

**Background:**

Patients with peanut allergy (PA) experience significant burden of illness, which impacts health‐related quality of life (HRQoL), particularly in adolescence. There is a paucity of research evaluating drivers of HRQoL scores.

**Methods:**

A prospective, online survey of adolescents with self‐reported, provider‐diagnosed PA completed from November 2018 to January 2019 was used to explore drivers of the real‐world impact of PA on HRQoL using the Pediatric Quality of Life Inventory 4.0 (PedsQL) and other measures. Univariate and multivariate analyses were used to identify potential factors associated with PedsQL scores and to understand the level of association.

**Results:**

A total of 102 adolescents were included. The final model included 10 variables: race, reported strict peanut avoidance, satisfaction with prophylaxis, moderate‐to‐severe reaction within the past 12 months, touching peanut as cause of most severe reaction, fear of reaction, age, gender, comorbidities, and daily life limitations. In total, three items were shown to be strong predictors of the PedsQL total score including cause of severe reaction was touching peanut (yes), level of agreement with avoiding peanut (completely agree), and satisfaction with prophylaxis (not very much/not at all).

**Conclusions:**

There is substantial heterogeneity in the impact of the burden of PA on PedsQL scores across patients. This indicates the importance of shared and individualized decision making for PA management to optimize outcomes and improve HRQoL.

## 1 INTRODUCTION

Peanut allergy (PA) is one of the most common food allergies in children and adolescents in the US.^1^, ^2^ Despite changes in management, including early introduction of peanuts to infants within the first year of life, the prevalence has remained consistent at approximately 2.2% of the US population.^1^, ^3^

PA represents a significant burden for patients and caregivers including risks of accidental exposure, severe reactions, and anaphylaxis, increased healthcare utilization, direct and indirect costs, and even mortality.^2^ The Peanut Allergy Burden Study (PABS) was a large survey study that assessed the real‐world burden of peanut allergies on patients and caregivers in the US. In an analysis of PABS, McCann et al. reported frequent healthcare resource utilization in children, adolescents, and adults including regular and unscheduled allergist appointments, general practitioner appointments, over the counter medication usage, and epinephrine autoinjector usage as well as substantial loss in productivity. Additionally, in the past 12 months, 56.9%–59.9% of adolescent and adult patients and caregivers reported at least one PA‐related emergency or urgent care visit, 36.3%–47.4% reported an overnight hospital admission, 37.2%–52.3% reported intravenous (IV) epinephrine use, and 26.5%–39.8% reported intubation in the past 12 months.^4^

While PA represents a societal burden, it also represents a burden on the individual due to dietary restrictions, precautions on reading food labels and eating at restaurants, exposure during travel, work, or school, and an emotional toll that may affect the patient or caregiver's health‐related quality of life (HRQoL).^2^ Published literature have reported significant impacts of PA on HRQoL measures in patients, parents, and siblings including impairment in the familial–social dimension in peanut allergic children and adolescents.^5^, ^6^ In an assessment of HRQoL from PABS data, Nowak‐Wegrzyn reported that PA patients and caregivers had worse HRQoL when compared to healthy populations in all domains of the survey and, when compared to a chronically ill population, reported worse overall HRQoL, psychosocial, emotional, and social functioning. Specifically, adolescents were reported experiencing a greater impact on overall health‐related HRQoL than adults due to the social burden of allergen avoidance and dietary restriction.^7^

Despite these results, there is a paucity of HRQoL data and studies specifically evaluating the experience of adolescents with PA. The goal of this analysis was to utilize PABS data to identify potential predictors of Pediatric Quality of Life Inventory 4.0 (PedsQL) scores in adolescents with PA to add to the literature base and improve management opportunities for these patients.

## 2 METHODS

### 2.1 Survey study design

PABS was a cross‐sectional survey employed to examine the real‐world impact of PA in peanut‐allergic adolescents and adults, as well as caregivers of peanut‐allergic children. This study is a sub‐analysis of the peanut‐allergic adolescent sample to identify potential real‐world predictors of PedsQL scores in this population. The study was reviewed and approved by the Solutions Institutional Review Board (IRB).^8^

### 2.2 Data collection and procedures

This was a prospective, online survey of adolescents with self‐reported, provider‐diagnosed PA. Participants were recruited through emails sent by Lightspeed, a market research company with a US panel of 1.3 million individuals, inviting them to participate in the survey. A screening questionnaire was used to determine eligibility with the inclusion and exclusion criteria described below. Eligible participants who completed the screening questions and met the inclusion criteria were invited to complete the online survey. Participants gave informed consent to be in the overall research panel and to voluntarily opt‐in to complete this survey. A caregiver's consent was also required for the adolescent to participate.

Data collection occurred from November 2018 through January 2019. The survey took approximately 20–30 min to complete, and consisted of demographic questions, questions about medical and treatment history related to PA, food allergy, and general HRQoL measures, mental, physical, and social well‐being, treatment satisfaction, family dynamics and activities, caregiver burden, healthcare resource utilization, and questions about productivity related to work, home activities, and school. Questions analyzed and reported in previous manuscripts (i.e., productivity questions and another HRQoL measure) will not be described in the current manuscript.^4^, ^7^ Study participants were compensated with “award points” that could be redeemed for gift certificates, merchandise, or cash.

### 2.3 Participants

PABS recruited three samples consisting of peanut‐allergic adolescents, peanut‐allergic adults, and caregivers of peanut‐allergic children. Inclusion criteria for each sample were previously published.^4^, ^7^ Since the current study aimed to identify potential factors associated with PedsQL scores in peanut‐allergic adolescents, only one sample was used in this analysis. Eligible peanut‐allergic adolescents met the following inclusion criteria: (1) between 13 and 17 years old; (2) self‐reported physician‐diagnosed PA; (3) use of medication or medical care to treat a PA reaction or patient/caregiver always carries emergency medication; (4) “agree completely” or “very much” that they avoid being around peanuts; and (5) English‐speaking US resident. Participating in a clinical study for PA treatment was allowed. For peanut‐allergic adolescents, recruitment quotas were set to ensure an approximate 50:50 male to female ratio. After gender quota was met, potential participants who met the inclusion criteria were excluded. The response rate was 97% after inclusion and exclusion criteria were applied.

### 2.4 Baseline demographics and medical history

Demographic information collected included age, gender, and race/ethnicity. Medical history questions pertained to the patient's age at the first‐ever allergic reaction to peanuts, age at which PA was diagnosed, method of diagnosis, severity of PA reactions experienced in the past 12 months and over their lifetime, symptoms during the last allergic reaction, and the worst lifetime allergic reaction, treatment history, and satisfaction with treatment.

### 2.5 Healthcare resource utilization

PABS assessed PA‐specific healthcare resource utilization through questions beginning with the prompt of: “Thinking about your medical care and allergic reactions due to peanut.” Participating peanut‐allergic adolescents were asked to report PA‐related healthcare resource utilization in the previous 12 months and over their lifetime through a variety of prompts.

### 2.6 PedsQL

The PedsQL questionnaire administered to peanut‐allergic adolescents is a validated, 23‐item self‐report instrument that measures HRQoL with developmentally appropriate self‐report forms for children (age 8–12) and adolescents (age 13–18), and proxy‐report forms for parents HRQoL in healthy children and adolescents, as well as those with acute and chronic health conditions.^9^, ^10^ The measure consists of Generic Core scales, which were designed to measure the core dimensions of health as delineated by the World Health Organization, as well as role (school) functioning.^11^ The PedsQL encompass four domains: Physical Functioning (eight items), Emotional Functioning (five items), Social Functioning (five items), and School Functioning (five items), as well as three summary scores: Physical Health Summary Score (eight items), Psychosocial Health Summary Score (15 items), and Total Scale Score (23 items). Each question was answered on a reverse‐scored 5‐point Likert scale (never, almost never, sometimes, often, almost always). Each sub‐scale and composite scale were transformed to a 0–100‐point scale so that higher scores indicated better HRQoL.

### 2.7 Statistical analysis

A total of 170 potential variables covering demographic information, medical and treatment history, and healthcare resource utilization were identified in PABS. A few summary variables were derived from existing variables: total number of food allergies (included allergy to egg, dairy, soy, tree nut, wheat, fish, and others), total number of reactions in last 12 months and in lifetime, total number of moderate or severe reactions in last 12 months and in lifetime, total number of discrete episodes of healthcare resource utilization in last 12 months and in lifetime (including number of regularly scheduled and unscheduled allergist appointments, appointments with general practitioner or other healthcare provider, emergency department or urgent care visits, overnight or longer hospital admissions, over‐the‐counter medication use, prescription or use of an epinephrine autoinjector, number of times patients received IV epinephrine, and number of times patient required intubation), total number of reported symptoms of most severe reaction and a categorical variable indicating the most worrisome PA symptom among gastrointestinal symptoms, respiratory symptoms, visible signs, and dizziness.

Given limited sample size and large number of potential variables, a multi‐phase approach was taken to refine the list of potential variables prior to running stepwise multivariate analysis. First, a univariate, qualitative assessment of distributions for all 170 potential variables was conducted to determine the level of correlation between individual variables and PedsQL scores via *t*‐tests for categorical variables or Pearson/Spearman correlation for continuous or ordinal variables. Variables with little or no correlation (i.e., *p* > 0.30) or no clinical justification for inclusion were excluded. During this step, correlations between variables were assessed for potential multicollinearity. For multi‐level variables, different strata were tested because of few responses in certain levels.

After the initial assessment, there were 25 variables identified for use in a stepwise regression which retained six variables in the final model with a statistical significance threshold of 0.15. Due to their clinical relevance, variables for age, gender, total number of inflammatory illnesses (rhinitis, allergies to other substances, uncontrolled or controlled asthma, chronic lung diseases, eczema, etc.), and daily life limitations were added back in to have the final multivariate model evaluating the relationship of each variable to the PedsQL scores, after accounting for the impact of the other variables. This same model was then run for each of the PedsQL domain scores to determine if there were any differences in relationships observed for the domains. All statistical analyses were conducted using SAS Enterprise Guide 8.3 (SAS Institute Inc., Cary, NC, USA).

## 3 RESULTS

**FIGURE 1:**
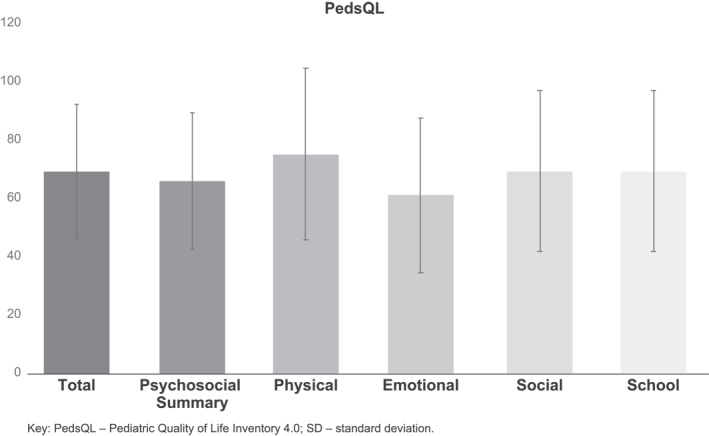
Mean (SD) self‐reported PedsQL scores of adolescents with peanut allergy (*N* = 102).

A total of 102 adolescents with PA completed PABS. The mean age was 14.7 years (standard deviation [SD]: 1.4), 55.9% were male, and 62.8% were white (Table 1). The mean (SD) PedsQL total score was 69.4 (23). Among the domains, Emotional Functioning (mean = 61.3, SD = 26.7) was scored the worst and Physical Functioning (mean = 75.4, SD = 29.4) was scored the best by peanut‐allergic adolescents. Of the two summary scores, adolescents rated Physical Health (mean = 75.4, SD = 29.4) higher than Psychosocial Health (mean = 66.2, SD = 23.4) (Figure 1).

**TABLE 1:** Demographic characteristics of peanut‐allergic adolescents.

Characteristic	*N* = 102
Age (years), mean (SD)	14.6 (1.3)
Gender, *n* (%)	
Male	57 (55.9%)
Female	45 (44.1%)
Hispanic, Latino, or Spanish ethnicity, *n* (%)	
Yes	21 (20.6%)
No	81 (79.4%)
Race, *n* (%)	
White	64 (62.8%)
Black/African American	19 (18.6%)
Asian	5 (4.9%)
American Indian or Alaska Native	2 (2.0%)
Mixed race	10 (9.8%)
Other	1 (1.0%)
Prefer not to answer	1 (1.0%)

### 3.1 Analytic model

**FIGURE 2:**
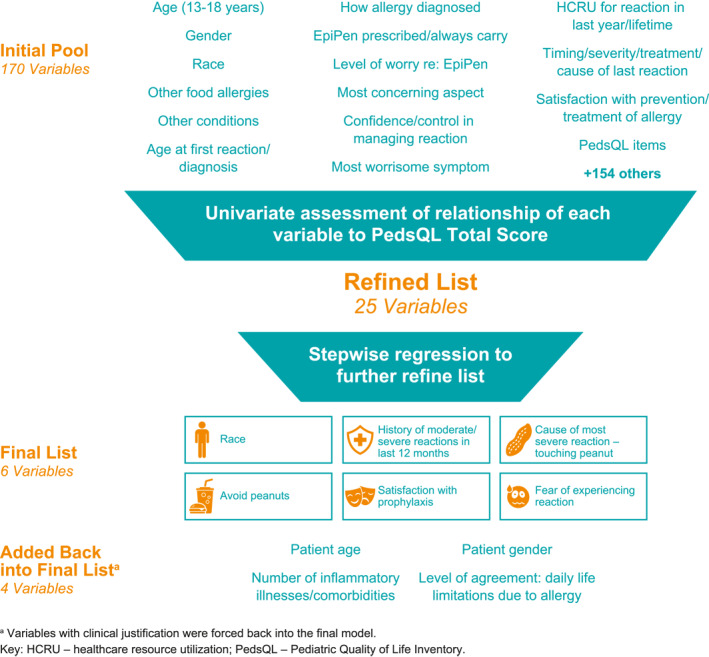
Selection of variables for the final model.

Of approximately 170 items collected within PABS, 25 items were at least weakly correlated with PedsQL total scores and included in a multivariate assessment (Figure 2). Stepwise regression resulted in a six‐variable model (adjusted R‐squared: 0.2997). Based on clinical justification, the final model also added four more variables including age, gender, comorbidities, and daily life limitations (adjusted R‐squared: 0.297) (Table 2).

**TABLE 2:** Adjusted R‐squared and beta coefficients for total and domain scores in the full model.

Variable	Response	Physical	Emotional	Social	School	Psychosocial	Total
Adjusted R‐squared	‐	0.237	0.142	0.296	0.210	0.310	0.297
Beta coefficients							
Age	‐	−1.045	−0.528	0.443	1.003	0.469	0.043
Female	‐	−0.792	1.619	4.451	1.832	2.651	1.301
Race	White	‐	‐	‐	‐	‐	‐
	Non‐white	3.333	7.735	5.128	3.983	4.964	5.076
Number of inflammatory illnesses	0	‐	‐	‐	‐	‐	‐
	1	−8.203	−0.590	−13.966^a^	−12.608^a^	−10.042^a^	−7.197
	2+	−5.947	−2.766	−9.135	−9.024	−7.282	−5.623
History of moderate/severe reaction in the last 12 months	Yes	−10.872^a^	−7.499	−4.861	−3.946	−6.235	−6.879
Touching peanut causes most severe reaction	Yes	−18.006^a^	−6.876	−13.280^a^	−12.469^a^	−11.980^a^	−12.945^a^
Avoid peanuts	Agree very much	‐	‐	‐	‐	‐	‐
	Agree completely	−12.502	−7.913	−18.198^a^	−18.841^a^	−15.217^a^	−13.336^a^
Daily life limitations	Not at all/not very/somewhat	‐	‐	‐	‐	‐	‐
	Very much/completely	−9.166	1.448	−9.675	−1.396	−4.776	−3.986
Satisfaction with prophylaxis	Not at all/not very/somewhat	−4.301	−9.588^a^	−16.681^a^	−7.530	−11.090^a^	−8.765^a^
	Very much/completely	‐	‐	‐	‐	‐	‐
Fear of experiencing reaction	Not at all	−20.342	−5.365	−15.569	−7.923	−7.176	−14.494
	Not very much	3.982	14.767	14.329	12.648	15.350^a^	9.031
	Somewhat	−5.121	6.750	8.189	9.399	9.444	2.740
	Very much	6.868	1.363	11.290	10.556	10.543	5.546
	Completely	‐	‐	‐	‐	‐	‐

^a^ Significant values (*p* < 0.05).

In total, three items were shown to be strong predictors of the PedsQL total score including cause of severe reaction was touching peanut (yes), level of agreement with avoiding peanut (completely agree), and satisfaction with prophylaxis (not very much/not at all). Other variables such as the number of moderate/severe reactions in the last 12 months and the number of co‐morbid allergic conditions were borderline significant in the total score PedsQL model and sporadically significant in the domain models, indicating a potential relationship. Applying the same regression model across individual PedsQL domain scores resulted in similar findings, with some variables moving in or out of statistical significance, as shown in Table 2.

## 4 DISCUSSION

Identifying drivers of HRQoL survey scores is imperative in transforming reported responses to actionable data that supports clinical decision making. In this study, survey responses to the PedsQL were strongly driven by three factors relating to the cause of severe reactions, the level of avoidance of peanuts, and dissatisfaction with PA prophylaxis. However, when the model was applied to individual patient scores, more heterogeneity in the drivers of results were observed, highlighting the complexity of successful management of adolescents with PA. These results are aligned with previous literature reporting a wide variety of HRQoL score drivers among different patient age groups, ethnicities, and countries of origin.^12^, ^13^

In a previous publication of PedsQL scores in adolescents in PABS, Nowak‐Wegrzyn reported that adolescents with PA had significantly worse total PedsQL scores and significantly worse scores in all domains when compared to a sample of healthy adolescents (*p* < 0.001). This included mean scores greater than 1 minimal important difference (MID) lower in all domains than the mean scores of healthy adolescents. Similarly, when compared to a chronically ill population of adolescents, those with PA reported significant worse total scores as well as significantly worse scores in the psychosocial, emotional, and social domains (*p* < 0.001). Mean total scores were greater than 1 MID lower for adolescents with PA when compared to chronically ill patients. This finding accentuates the need for effective interventions to improve HRQoL in adolescents with PA.^7^

The impact of PA on HRQoL is consistently reported across the literature. DunnGalvin et al. conducted semi‐structured interviews in 24 children, 39 teenagers, and 44 caregivers with moderate to severe PA in eight European countries. Themes identified included impacts on social, emotional, work, and relationships as well as themes relating to coping and control. Drivers of these themes included social attitudes and support, child–caregiver relationship, and coping strategies used. Importantly, they concluded the burden of PA does not necessarily correlate with the severity of PA, substantiating the need for individualized patient management.^13^

Avery et al. conducted a survey using two disease‐specific HRQoL questionnaires in 20 children with PA and 20 children with insulin‐dependent diabetes mellitus (IDDM) and collected photographs relating to their disease over a 24‐h period. Children with PA reported significantly worse HRQoL than patients with IDDM in both questionnaires (*p* = 0.004 and *p* ≤ 0.001) and reported more fear of an adverse event and eating, particularly away from home. Authors noted children with PA were aware of the potential for a fatal event due to their condition.^14^ Similarly, Roy et al. assessed 51 families of children with confirmed PA through questionnaires regarding child anxiety, parenting stress, and HRQoL. Reported results showed child anxiety and parenting stress significantly predicted parent proxy reporting of their child's HRQoL; other drivers of HRQoL identified included child anxiety, parenting stress, length of diagnosis, and experiencing an epinephrine shot.^15^

Finally, King et al. examined the impact of PA on HRQoL in children with PA, their parents, and older siblings representing 46 families using PedsQL, the World Health Organization QoL scale (brief version) (WHOQOL‐BREF), Spence child anxiety scale (SCAS), state‐trait anxiety inventory (STAI), and perceived stress scales. Mothers reported significantly more anxiety and stress (*p* < 0.05 and *p* < 0.001, respectively) as well as significantly worse psychological and physical HRQoL (*p* < 0.01 and *p* < 0.05, respectively) than fathers and reported a greater impact on HRQoL for their child with PA than both fathers and the child with PA themselves (*p* < 0.01). Children with PA had significantly worse physical HRQoL, HRQoL within school, general HRQoL, and greater separation anxiety than their siblings (*p* < 0.05, *p* < 0.01, *p* < 0.05, *p* < 0.05, respectively).^5^

Taken together, the results of this and other published studies highlight the need for consideration of improvement in HRQoL as an important outcome of prevention and treatment of PA. In 2017, the National Institute of Allergy and Infectious Diseases sponsored a panel to update the guidelines for prevention of PA in the US, which included the addition of peanut‐containing foods earlier in life for high‐risk populations.^16^ However, the standard of care for the management of PA remained dietary restriction until the emergence of immunotherapy, allowing for increasing doses of peanut to children with PA with the goal of allowing safe exposure to peanut.^17^, ^18^ Based on the results of the PALISADE trial, the Food and Drug Administration (FDA) approved an oral immunotherapy (OIT) product, peanut (*Arachis hypogaea*) allergen powder‐dnfp, in 2020 to mitigate allergic reactions after accidental peanut exposure in peanut‐allergic individuals aged 4–17 years^19^, ^20^, ^21^ Other emerging management options include epicutaneous immunotherapy (EPIT), which consists of a peanut patch on the surface of the skin and is under investigation in multiple clinical trials,^22^, ^23^, ^24^ as well as sublingual immunotherapy, DNA vaccines, and adjuvant‐enhanced immunotherapy.^17^

As these novel therapies come to market, it is important to assess their impact on HRQoL. HRQoL was evaluated in two clinical trials of EPIT (PEPITES and PEOPLE) using the food allergy quality of life questionnaire (FAQLQ) in children with PA that received EPIT or placebo or their parents. Results in 152 children and 305 parents were reported; EPIT treatment was associated with significant global and domain‐specific FAQL improvements when compared to the placebo group.^25^ Similarly, treatment with OIT has been associated with improved HRQoL in both patients and caregivers.^26^, ^27^ In the present reported study, results showed a correlation between lack of satisfaction with current therapy (i.e., avoidance) and poorer HRQoL, and thus it stands to reason that novel therapies may improve HRQoL. Understanding the potential HRQoL outcomes associated with emerging PA therapies will help describe the full impact of these management strategies and inform shared provider, patient, and/or caregiver decision making.

### 4.1 Limitations

This survey relied on self‐reported measures, which may introduce several forms of bias into the results. Given that inclusion criteria specified the enrollment of only English‐speaking US residents, the results might have been limited by the overall diversity in the sample pool. However, it is notable that the sample was racially diverse, with almost 40% of patients being non‐White. To our knowledge, this is the first study to capture such a diverse patient population in this space. Next, because patients had to require either medication or medical care because of a peanut reaction, results may not represent all individuals with PA, particularly those where avoidance of peanuts is a successful strategy or who may not be sensitive to very small amounts of peanut. Finally, there are specific instruments that exist evaluating food allergy in adolescents, such as the FAQL‐teen,^28^ but we utilized the PedsQL instrument as an instrument that would enable a comparison of the responses of adolescents with PA to those of their peers without PA.

## 5 CONCLUSIONS

There is substantial heterogeneity in the impact of the burden of PA on PedsQL scores across patients. This indicates the importance of shared decision making for PA management to optimize outcomes and improve HRQoL.

## AUTHOR CONTRIBUTIONS

**William A. McCann:** conceptualization; methodology; supervision; writing, review, and editing. **Anna Nowak‐Wegrzyn:** conceptualization; methodology; supervision; writing, review, and editing. **Steven L. Hass:** conceptualization; methodology; validation; formal analysis; investigation; supervision; writing, review and editing; visualization. **Danmeng Huang:** conceptualization; methodology; validation; writing, review, and editing. **Sarah M. Donelson:** conceptualization; methodology; writing, review, and editing; funding acquisition.

## CONFLICT OF INTEREST STATEMENT

William A. McCann, Anna Nowak‐Wegrzyn, Steven L. Hass are paid consultants for Aimmune Therapeutics; Danmeng Huang was an employee of Xcenda, LLC at the time this research was conducted; Sarah M. Donelson was an employee of Aimmune Therapeutics at the time this research was conducted.

## Data Availability

The datasets used and/or analyzed during the current study are available from the corresponding author on reasonable request.
